# Association between severe childhood infections and subsequent risk of OCD is largely explained by shared familial factors

**DOI:** 10.1136/bmjment-2024-301203

**Published:** 2024-10-25

**Authors:** Josep Pol-Fuster, Ralf Kuja-Halkola, Lorena Fernández de la Cruz, Isabell Brikell, Zheng Chang, Brian M D’Onofrio, Henrik Larsson, Paul Lichtenstein, Jan C Beucke, Elles De Schipper, David Mataix-Cols

**Affiliations:** 1 Centre for Psychiatry Research, Department of Clinical Neuroscience, Karolinska Institutet & Stockholm Health Care Services, Region Stockholm, Stockholm, Sweden; 2 Department of Medical Epidemiology and Biostatistics, Karolinska Institutet, Stockholm, Sweden; 3 Department of Global Public Health and Primary Care, University of Bergen, Bergen, Norway; 4 Department of Biomedicine, Aarhus University, Aarhus, Denmark; 5 Department of Psychological and Brain Sciences, Indiana University, Bloomington, Indiana, USA; 6 School of Medical Sciences, Örebro University, Örebro, Sweden; 7 Institute for Systems Medicine, Department of Human Medicine, MSH Medical School Hamburg, Hamburg, Germany; 8 Department of Clinical Sciences, Lund University, Lund, Sweden

**Keywords:** Child & adolescent psychiatry, PSYCHIATRY

## Introduction

Obsessive-compulsive disorder (OCD) is only moderately heritable^1 2^, but surprisingly little is known about potential environmental risk factors contributing to its aetiology.^3 4^

A potential environmental risk factor that has gathered considerable attention is infections, hypothesised to be causally related to OCD through postinfectious autoimmune processes. While some population-based studies have shown an association between infections and OCD, a common limitation of these studies is the lack of control for familial confounding, making causal inference impossible.^5 6^ A recent genetically informative population-based study concluded that the association between severe childhood infections and subsequent OCD may be largely explained by familial confounding.^6^ However, the coverage of infections was incomplete because this study restricted the exposure period to the first 3 years of life and only focused on very severe infections requiring hospitalisation.^7^ The current sibling-controlled cohort study aimed to further investigate the association between childhood infections recorded before age 12 years (the typical age of onset of childhood OCD) and the subsequent risk of OCD, including infections treated in both inpatient and specialised outpatient settings.

## Methods

We identified a cohort of 2226 386 individuals born in Sweden between 1 January 1987 and 31 December 2008 with information on both biological parents. Considering that the minimal age for the outcome was age 6 years, individuals who emigrated or died before age 6 years were excluded (n=60 026). This cohort was followed from birth until the date of OCD diagnosis, emigration, death or end of the follow-up (31 December 2020), whichever came first.

Childhood infections were defined as any record of infection in the National Patient Register (NPR), including both inpatient (from 1987) and specialised outpatient settings (from 2001), occurring within an individual’s first 12 years of life (see International Classification of Diseases (ICD) codes in table 1). We also analysed bacterial and viral infections separately. OCD was defined as the first recorded instance of a diagnosis (ICD-9: 300D; ICD-10: F42) in the NPR after the age of 6 years, hence minimising the risk of diagnostic misclassification.^1^

**Table 1:** Distribution of study cohort characteristics

		No record of OCD (n=2201 394) *n (%*)	OCD (n=24 992) *n (%*)
Sex	Male	1133 683 (51.5)	10 488 (42.0)
	Female	1067 711 (48.5)	14 504 (58.0)
Birth year	1987–1991	554 395 (25.2)	7734 (31.0)
	1992–1996	522 442 (23.7)	6863 (27.5)
	1997–2002	522 552 (23.7)	6687 (26.8)
	2003–2008	602 005 (27.4)	3708 (14.8)
Any infection^*†‡^		608 851 (27.7)	7059 (28.2)
Bacterial infections^†^		391 302 (17.8)	4472 (17.9)
Viral infections^‡^		336 176 (15.3)	3939 (15.8)

*Swedish International Classification of Diseases, 9th edition (ICD-9) codes: 466A 466B 478B 478C. Swedish International Classification of Diseases, 10th edition (ICD-10) codes: J200 J201 J202 J390 J391.

†Swedish ICD-9 codes: 001 002 003 004 005 008A 008B 008C 008D 008E 008F 01 02 030 031 032 033 034B 035 036 037 038 039 041 073 076 077A 078D 078J 080 081 082 083 087 091 092 093 094 095 096 097 098 099A 099B 099C 100 101 102 103 104 320 324 325 326 382 390 391 475 481 482 510 513 540 541 542 590 595 597A 599A 614 615 616 646F 646G 68 711A 711E 790H. Swedish ICD-10 codes: A00 A01 A02 A03 A04 A05 A1 A2 A30 A31 A32 A34 A35 A36 A37 A38 A39 A4 A51 A52 A53 A54 A55 A56 A57 A58 A65 A66 A67 A68 A69 A7 B95 B96 G00 G01 G042 G050 G06 G07 G08 G09 H66 H670 I00 I01 J13 J14 J15 J170 J200 J201 J202 J36 J390 J391 J85 J86 K35 K36 K37 L0 M00 M010 M011 M012 M013 N10 N11 N12 N30 N340 N390 N7 O23.

‡Swedish ICD-9 codes: 008H 008J 008K 008L 008M 008W 045 046 047 048 049 05 06 070 071 072 074 075 077B 077C 077D 077E 077W 077X 078A 078B 078E 078F 078G 078H 078W 079 321E 321H 480 487 711F 790W. Swedish ICD-10 codes: A08 A8 A9 B0 B1 B2 B30 B33 B34 B97 G020 G051 H671 J10 J11 J12 J171 J203 J204 J205 J206 J207 J210 M014 M015.

OCD, obsessive-compulsive disorder.

To examine the association between childhood infections and the subsequent risk of OCD, we performed Cox proportional hazards regression analyses with first infection as time-varying exposure and age as the underlying time scale to estimate hazard ratios (HRs) and 95% confidence intervals (CIs). Then, we conducted conditional Cox proportional hazards regression analyses comparing exposed full siblings with their unexposed counterparts with each family as a strata (ie, discordant siblings design). This comparison automatically excludes confounding from all shared environmental factors and a substantial proportion (50%) of genetic factors.

All models were adjusted for sex and birth year (using the categories in table 1). Robust standard errors were applied in all analyses to address familial clustering. Data were analysed from 1 January 2024 to 1 May 2024. Statistical analyses were conducted in SAS (V.9.4; SAS Institute) and R using the Survival package.

## Results

Descriptive characteristics are presented in table 1. Out of the 2226 386 individuals in the cohort, 24 992 were diagnosed with OCD. The median age at first OCD diagnosis was 18.16 years (IQR: 7.82). Within this population, 1572 545 individuals who had at least one sibling were included in the sibling analyses, comprising 691 270 clusters.

**Figure 1:**
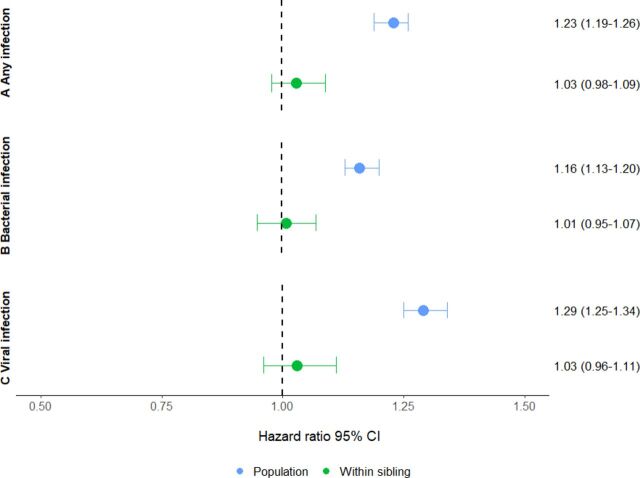
Association between any childhood infections (A), bacterial infections (B), viral infections (C) and obsessive-compulsive disorder in the population cohort (blue) and in the sibling cohort (green).

Individuals exposed to any infection during their first 12 years of life had a 23% higher risk of being diagnosed with OCD, compared with unexposed individuals (HR 1.23; 95% CI 1.19 to 1.26) (figure 1). This association was also found for both bacterial (HR 1.16; 95% CI 1.13 to 1.20) and viral (HR 1.29; 95% CI 1.25 to 1.34) infections. However, when comparing clusters of discordant siblings, these associations attenuated to the null and were no longer statistically significant for either of the variables: any infection (HR 1.03; 95% CI 0.98 to 1.09), bacterial (HR 1.01; 95% CI 0.95 to 1.07) and viral (HR 1.03; 95% CI 0.96 to 1.11) (figure 1).

## Discussion

In this population-based birth cohort, childhood infections were significantly associated with a higher risk of OCD. However, our sibling analysis suggested that the observed risk was strongly influenced by familial factors, rather than representing a direct causal effect. These results both support and extend previous research^6^ benefiting from a better register coverage, an extended exposure period and the inclusion of milder infections. Further research is needed to clarify the specific influence of genetic and/or environmental factors on the observed association.

Among the study’s strengths are the population-based prospective design and the reliability and validity of OCD codes in the NPR.^8^ Some limitations include incomplete coverage of outpatient diagnoses before 2001. Additionally, individuals with mild infections and/or OCD who do not seek help or are managed in primary care are not captured by the NPR.
